# The Role of Nrf2 Signaling Pathway in *Eucommia ulmoides* Flavones Regulating Oxidative Stress in the Intestine of Piglets

**DOI:** 10.1155/2019/9719618

**Published:** 2019-09-02

**Authors:** Dingfu Xiao, Daixiu Yuan, Bihui Tan, Jing Wang, Yanhong Liu, Bie Tan

**Affiliations:** ^1^College of Animal Science and Technology, Hunan Agricultural University, Changsha, 410128 Hunan, China; ^2^Department of Medicine, Jishou University, Jishou, 416000 Hunan, China; ^3^National Engineering Laboratory for Pollution Control and Waste Utilization in Livestock and Poultry Production, Key Laboratory of Agro-Ecological Processes in Subtropical Region, Institute of Subtropical Agriculture, Chinese Academy of Sciences, Changsha, 410125 Hunan, China; ^4^Department of Animal Science, University of California Davis, Davis, 95616 CA, USA

## Abstract

*Eucommia ulmoides* flavones (EUF) have been demonstrated to alleviate oxidative stress and intestinal damage in piglets, but their effect target is still poorly understood. NF-E2-related factor 2 (Nrf2) pathway plays a very important role in the defense mechanism. This study was designed to investigate the regulation of EUF on the Nrf2 pathway and inhibition of Nrf2 on oxidative stress in the intestine of piglets. An in vivo study was conducted in weaned piglets treated with basal diet, basal diet+diquat, and 100 mg/kg EUF diet+diquat for 14 d to determine Nrf2 and Keap1 protein expressions, as well as downstream antioxidant gene mRNA expression. An in vitro study was performed in a porcine jejunal epithelial cell line to investigate the effect of inhibiting Nrf2 on cell growth and intracellular oxidative stress parameters. The results showed that the supplementation of EUF decreased the oxidized glutathione (GSSG) concentration and the ratio of GSSG to glutathione (GSH) but increased the protein expressions of nuclear Nrf2 and Kelch-like ECH-associated protein 1 (Keap1) as well as mRNA expression of *heme oxygenase 1* (*HO-1*), *NAD(P)H:quinone oxidoreductase 1* (NQO-1), and *glutamate cysteine ligase catalytic subunit* (*GCLC*) in the small intestinal mucosa of diquat-challenged piglets. When Nrf2 was inhibited by using ML385, cell viability, cellular antioxidant activities, expressions of nuclear Nrf2 and Keap1 protein, and downstream antioxidant enzyme (*HO-1*, *NQO-1*, and *GCLC*) mRNA were decreased in paraquat-treated enterocytes. These results showed that the Nrf2 signaling pathway played an important role in EUF-regulating oxidative stress in the intestine of piglets.

## 1. Introduction

The intestinal tract of piglets is not fully developed and highly susceptible to stress due to its special vascular anatomical structure and convective oxygen exchange mechanism [1]. Oxidative stress is a key factor in the occurrence and development of intestinal injury [2]. Stress can induce the production of a large number of toxic reactive oxygen species (ROS) metabolites in the enterocytes and affect the stability of DNA and RNA as well as the activities of enzymes, resulting in intestinal mucosal damage [3]. Yin et al. found that early weaning at the age of 14 d damaged the oxidation balance of piglets, and the activities of superoxide dismutase (SOD) and glutathione peroxidase (GSH-Px) in plasma were significantly reduced, especially at 3 days after weaning [4]. Wang et al. also found that 21-day-old weaning could reduce glutathione (GSH) content by 25% and increase the ratio of oxidized glutathione (GSSG) to GSH by 59% in the jejunum of piglets [5]. Because of the rich xanthine oxidase in the intestinal tissue, the oxidative damage in the mucosal cells is more significant, and oxidative damage occurs the earliest, while the recovery is the slowest [6]. Long-term stress will further lead to inflammatory bowel disease (IBD), including ulcerative colitis and Crohn's disease. In the inflammatory bowel disease model, neutrophils were activated and released a large number of oxygen metabolites and proteases, resulting in the intestinal tissue damage [7].

Flavonoids have been reported to exhibit strong antioxidant activities that could directly scavenge free radicals and inhibit ROS, nitric oxide, and proinflammatory cytokine productions [8]. The previous study has indicated that dietary supplementation with *Eucommia ulmoides* flavones (EUF) alleviated the growth performance impairment, oxidative stress, inflammatory response, and intestinal damage induced by diquat in piglets [9]. However, the preciseness of targets of EUF-regulating oxidative stress in porcine enterocytes still needs to be elucidated.

Most of the flavonoids are poorly absorbed through the gut barrier [10], so the intestine is the major site of antioxidant defense afforded by flavonoids [11]. NF-E2-related factor 2 (Nrf2) is a key factor in the oxidative stress response and highly expressed in the gastrointestinal tract. It plays an important role in mediating oxidative stress in the small intestine and stomach [12, 13]. If Nrf2 is disabled or absent, the expression level of downstream antioxidant enzymes is reduced, and the toxicity of oxidative stress cannot be resisted, leading to cell dysfunction, apoptosis, or necrosis. An activated Nrf2 signaling pathway can inhibit ubiquitin-mediated degradation of Nrf2 protein and enhance the transcriptional activity of Nrf2 protein [14]. Many polyphenols can induce antioxidant response element (ARE) activation and enhance Nrf2 expression or nuclear translocation [15]. Therefore, the present study was conducted to investigate the regulation of EUF on the Nrf2 pathway in the intestine by using a diquat-induced oxidative stress piglet model. Meanwhile, a specific inhibitor ML385 was used to inhibit Nrf2 and investigate its effects on cellular antioxidant activities and downstream antioxidant enzyme mRNA expression in paraquat-treated enterocytes.

## 2. Materials and Methods

### 2.1. Animals and Experimental Design

The animal experiments were approved by the Institutional Animal Care and Use Committee of Hunan Agricultural University, Hunan, China.

A total of 24 piglets (Duroc×Landrace×Large Yorkshire) weaned at 21 days were randomly assigned to receive 1 of 3 treatments with 8 replicate pens/treatment: basal diet, basal diet+diquat, and 100 mg/kg EUF diet+diquat, respectively. The basal diet was formulated to meet the nutrient requirements for weanling piglets, and the dose of 100 mg/kg EUF was based on the results showed in the previous study [9]. EUF powder that contained 83.61% total flavones was prepared at the Department of Medicine, Jishou University (Jishou, Hunan, China), which has been used in the previous study by Yuan et al. [9]. The piglets were individually housed in an environmentally controlled nursery with hard plastic slatted flooring and had free access to feed and water. After the 7-day adaptation period, piglets were fed their respective diets 3 times per day for a 14 d period. On day 7 after the initiation of treatment, the piglets were injected intraperitoneally with diquat at 8 mg/kg BW or the same amount of sterilized saline, respectively. On day 14, all piglets were slaughtered and intestinal samples from the jejunum and ileum were collected and immediately snap-frozen in liquid nitrogen and stored at –80°C for further analysis.

### 2.2. Cell Culture

A porcine jejunal epithelial cell line, IPEC-J2 cells, was cultured with high-glucose (25 mM) Dulbecco's modified Eagle's medium (DMEM-H) (HyClone) containing 10% fetal bovine serum (Gibco) and 1% antibiotic solution (P/S; Sigma) at 37°C in a 5% CO_2_ incubator. Cells were grown to 90% confluence and treated with the following medium for an additional 12 h: (1) control, DMEM-H medium; (2) PQ, DMEM-H medium with 70 *μ*M paraquat (Sigma); and (3) ML385, DMEM-H medium with 1.9 *μ*M specific Nrf2 inhibitor ML385 (Selleckchem). The lethal dose 50 of 70 *μ*M paraquat and 1.9 *μ*M ML385 was obtained from the cell proliferation results.

### 2.3. Cell Viability and Cytotoxicity Assays

Cell viability was determined by using the Cell Counting Kit-8 (CCK-8, Dojindo Molecular Technologies Inc., Rockville, MD, USA). Lactate dehydrogenase (LDH) release was analyzed to evaluate the cytotoxicity using the LDH Cytotoxicity Assay Kit (Beyotime Institute of Biotechnology, China). The absorbance was measured using a microplate reader (TECAN, Männedorf, Switzerland) at 450 nm and 490 nm, respectively [16, 17].

### 2.4. Cellular Antioxidant Activity (CAA) Assay

After PQ and ML385 treatment, cells cultured in black 96-well plates (with a transparent bottom) were aspirated and then incubated in the dark with 10 *μ*mol/L 2′,7′-dichlorofluorescein diacetate (DCFH-DA) solution (Beyotime, China) at 37°C for 30 mins. After washing for three times by the serum-free medium, the cells were treated with 100 *μ*L of 600 mM 2,2′-Azobis (2-methylpropionamidine) dihydrochloride (Sigma, St. Louis, MO) solution. Dynamic DCF fluorescence was measured using a microplate reader (TECAN, Männedorf, Switzerland) with excitation at 488 nm and emission at 525 nm. After subtraction of the blank, the area under the curve (AUC) for fluorescence versus time was integrated to calculate the CAA value of each sample as follows:

(1) $$\begin{array}{c} \text{CAA}\left(\text{unit}\right)=\left\{1-\frac{\int \text{SA}}{\int \text{CA}}\right\}\times 100, \end{array}$$

where SA is the area of the sample and CA is the integrated area in the control curve (that generated from control cells treated with DCFH-DA and oxidant) [18].

### 2.5. Intestinal and Intracellular Oxidative Stress Parameter Measurements

Intestinal SOD activity and malondialdehyde (MDA) concentrations were analyzed by the xanthine oxidase-xanthine reaction method and 2-thiobarbituric acid method, respectively, using SOD and MDA assay kits (Nanjing Jiancheng, Nanjing, China) according to the manufacturer instructions.

Intestinal samples and cell samples were sonicated and deproteinated, respectively; then, supernatants and cell lysate were used to analyze the concentrations of GSSG and total GSH with a GSH and GSSG assay kit (Beyotime Institute of Biotechnology, China) according to the manufacturer's instructions. The results were normalized to the total protein content, and the ratio of GSSG to GSH was calculated.

### 2.6. Western Blot

Nuclear and cytosolic proteins from intestinal and cell samples were extracted by using a Nuclear-Cytosol Extraction Kit (Applygen, Beijing, China) according to the manufacturer's instructions. Nuclear and cytosol extractions were stored at –80°C for Nrf2 protein analysis, respectively. The protein expression levels of *β*-actin, Kelch-like ECH-associated protein 1 (Keap1), nuclear Nrf2, and cytosol Nrf2 were determined as previous descriptions [16]. The following antibodies were used for protein quantification: *β*-actin (1 : 4000, Proteintech), Keap1 (1 : 100, Santa Cruz), and Nrf2 (1 : 500, Santa Cruz). Data are expressed by the relative values of piglets fed the basal diet or control cells.

### 2.7. Real-Time PCR

Total RNA was extracted in cells and intestinal samples with a TRIzol reagent (Invitrogen, Carlsbad, CA, USA), and the cDNA was reverse-transcribed from eluted RNA using the first strand cDNA synthesis kit (Fermentas) according to the manufacturer's instructions. The real-time quantitative PCR for *β-actin*, *heme oxygenase 1* (*HO-1*), *NAD(P)H:quinone oxidoreductase 1* (*NQO-1*), *glutamate cysteine ligase catalytic subunit* (*GCLC*), and *glutamate cysteine ligase modifier subunit* (*GCLM*) was carried out using the following primers to amplify genes: *β-actin* (F) 5′-GGACCT GAC CGA CTA CCT CA-3′, (R) 5′-CAC AGC TTC TCCTTG ATG TCC-3′; *HO-1* (F) 5′-GTCCTTGTACCACATCTACGA-3′, (R) 5′-CCTTCTGAGCAATCTTCTTG-3′; *NQO-1* (F) 5′-TTTGAAGAGGAGAGGATGG-3′, (R) 5′-ATGGCAGCGTATGTGTAAG-3′; *GCLC* (F) 5′-GTAAGTCCCGATACGATTCA-3′, (R) 5′-TCTACTCTCCACCCAATGTC-3′; and *GCLM* (F) 5′-CCGATGAAAGAGAAGAAATG-3′, (R) 5′-ACACAGCAAGAGGCAAGAT-3′. The comparative threshold cycle (Ct) value method was employed to quantitate the expression levels for target genes relative to those for the *β*-actin. The data were expressed as the relative values of piglets fed the basal diet or control cells.

### 2.8. Statistical Analysis

The data were subjected to ANOVA analysis using SPSS 17.0 software (SPSS Inc., Chicago, IL, USA). The differences among treatments were evaluated using Tukey's test. Probability values < 0.05 were taken to indicate statistical significance.

## 3. Results

### 3.1. Small Intestinal Mucosal Concentrations of SOD, MDA, GSH, and GSSG

Exposure of diquat decreased SOD activity in the jejunal mucosa but increased GSSG concentration and the ratio of GSSG to GSH in the jejunal and ileal mucosa of piglets fed with basal diet (*p* < 0.05). In the diquat-challenged piglets, the supplementation of EUF increased MDA concentration in the jejunal mucosa but decreased GSSG concentration and the ratio of GSSG to GSH in the jejunal and ileal mucosa (*p* < 0.05). There were no differences in SOD activity as well as the concentrations of MDA and GSH in the jejunal and ileal mucosa of piglets between basal diet and EUF diet+diquat treatments (*p* > 0.05) (Table 1).

### 3.2. Protein Expression of Nrf2, Keap1, and mRNA Expression of Antioxidant Enzyme in the Small Intestine

**Figure 1:**
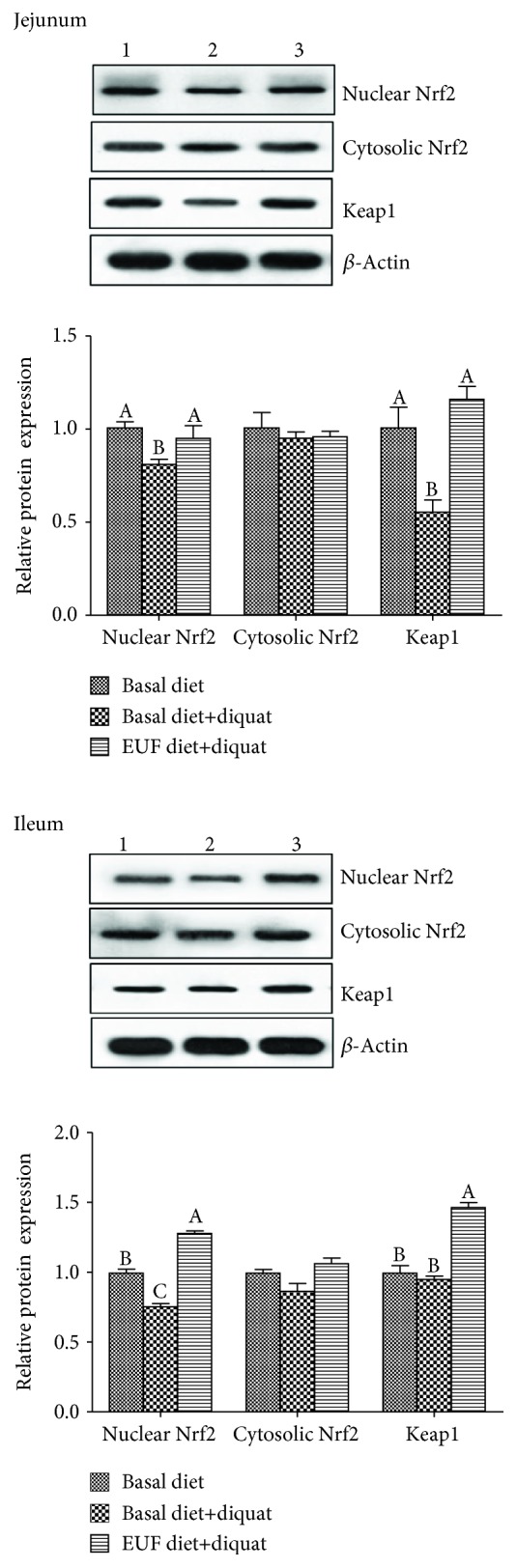
The representative western blot images (1: basal diet, 2: basal diet+diquat, and 3: EUF diet+diquat) and relative protein expressions of Nrf2 and Keap1 in the jejunum and ileum of piglets. Piglets were treated with basal diet, basal diet+diquat, and 100 mg/kg EUF diet+diquat, respectively, for 14 days. Data expressed as means ± SEM, *n* = 8. Different letters (A, B, and C) indicate significant differences (*p* < 0.05).

In the jejunum, diquat exposure decreased the protein expression of nuclear Nrf2 and Keap1, as well as *NQO-1* mRNA abundance (*p* < 0.05). However, EUF addition to the diquat-challenged piglets increased nuclear Nrf2 and Keap1 protein expression and mRNA expression of *HO-1*, *NQO-1*, and *GCLC* (*p* < 0.05). In the ileum, compared with basal diet treatment, the nuclear Nrf2 protein expression as well as *HO-1*, *NQO-1*, and *GCLC* mRNA abundances in piglets of basal diet+diquat treatment was decreased, but the protein expression of Nrf2 and Keap1 as well as *HO-1* mRNA expression in EUF diet+diquat-treated piglets was increased (*p* < 0.05). There was no difference in cytosolic Nrf2 protein expression both in the jejunum and ileum among three treatments (*p* > 0.05) (Figure 1 and Table 2).

### 3.3. Cell Viability and Cytotoxicity of the IPEC-J2 Cells

**Figure 2:**
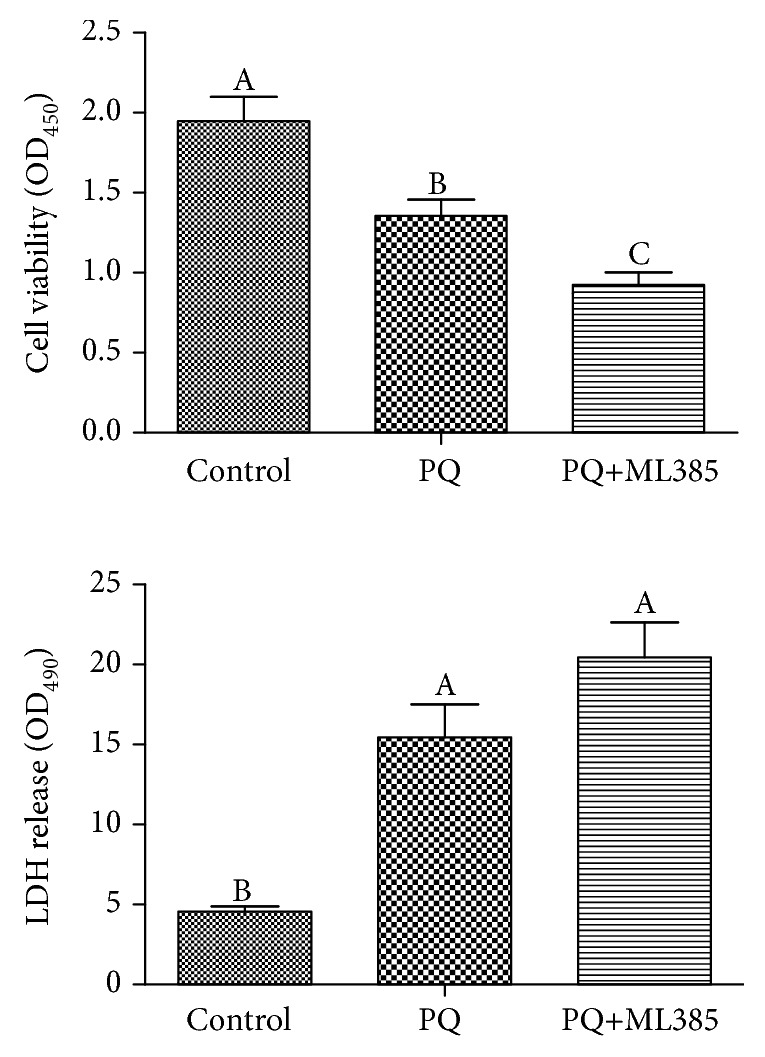
Cell viability and cytotoxicity. IPEC-J2 cells were treated with DMEM-H medium (control) or with 70 *μ*M paraquat (PQ) or 1.9 *μ*M specific Nrf2 inhibitor (ML385) for 12 h. Cell viability was measured using the Cell Counting Kit-8 from Dojindo Molecular Technologies. Cytotoxicity was determined by lactate dehydrogenase (LDH) release using the LDH Cytotoxicity Assay Kit from Beyotime Institute of Biotechnology. Data expressed as means ± SEM; *n* = 6 independent experiments. Different letters (A, B, and C) indicate significant differences (*p* < 0.05).

Compared with control cells, paraquat treatment decreased cell viability and increased LDH release, and these effects were strengthened by Nrf2 inhibition using ML385 (*p* < 0.05) (Figure 2).

### 3.4. Cellular Antioxidant Activities and Intracellular Oxidation State of GSH

**Figure 3:**
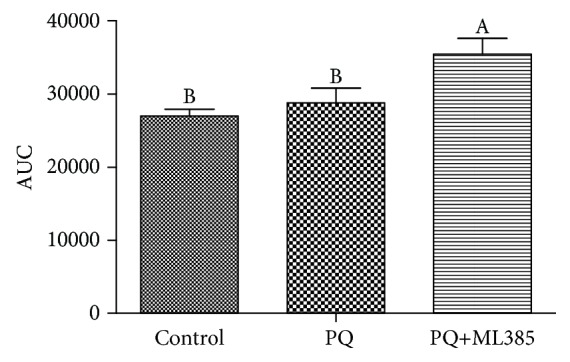
Cellular antioxidant activities. IPEC-J2 cells were treated with DMEM-H medium (control) or with 70 *μ*M paraquat (PQ) or 1.9 *μ*M specific Nrf2 inhibitor (ML385) for 12 h. The total area under the curve (AUC) was computed using the trapezoid rule based on kinetic fluorescence data. The lower AUC indicated greater cellular antioxidative activity. Data expressed as means ± SEM; *n* = 6 independent experiments. Different letters (A, B) indicate significant differences (*p* < 0.05).

**Figure 4:**
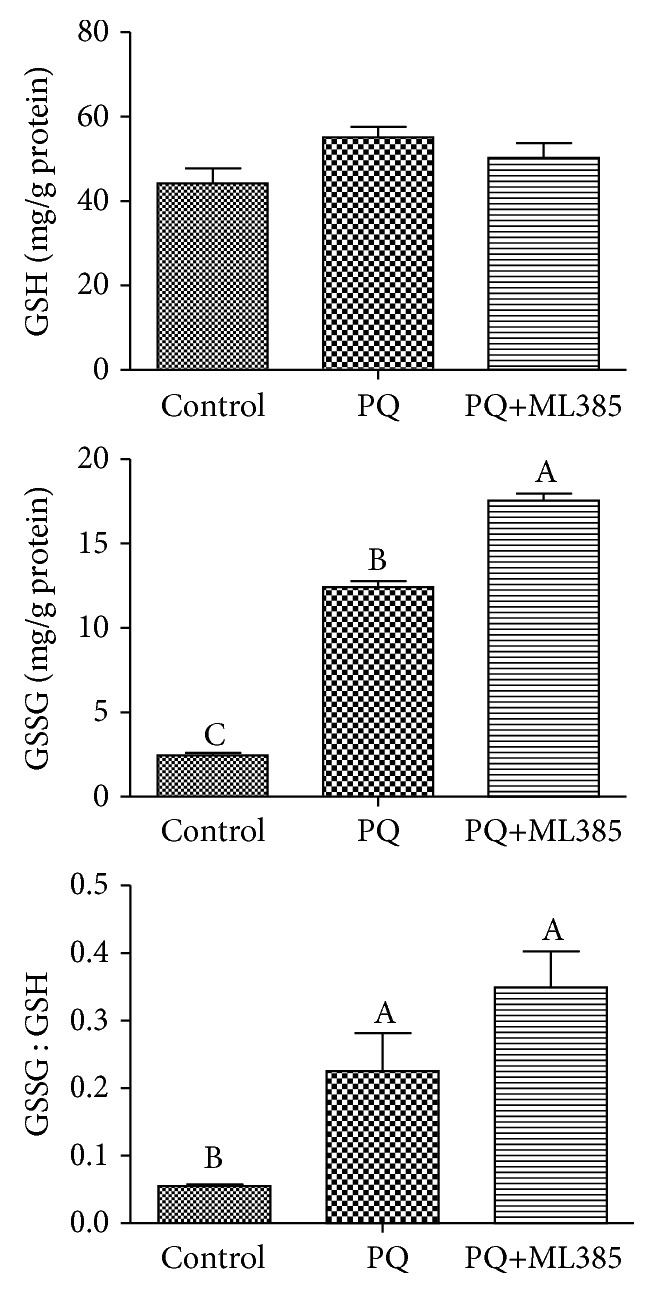
Intracellular glutathione (GSH) and oxidized glutathione (GSSG) concentrations. IPEC-J2 cells were treated with DMEM-H medium (control) or with 70 *μ*M paraquat (PQ) or 1.9 *μ*M specific Nrf2 inhibitor (ML385) for 12 h. The concentrations of GSH and GSSG were determined using a GSH and GSSG assay kit from Beyotime Institute of Biotechnology. The ratio of GSSG to GSH was calculated. Data expressed as means ± SEM; *n* = 6 independent experiments. Different letters (A, B, and C) indicate significant differences (*p* < 0.05).

AUC that negatively correlated with cellular antioxidant activities in the cells of PQ+ML385 treatment was higher (*p* < 0.05) than that of the control and PQ treatments (Figure 3). Both paraquat and ML385 treatments increased (*p* < 0.05) intracellular GSSG concentration and the ratio of GSSG to GSH, but there was no difference in total GSH content among three treatments (*p* > 0.05) (Figure 4).

### 3.5. Protein Expression of Nrf2, Keap1, and mRNA Expression of Antioxidant Enzyme in the IPEC-J2 Cells

**Figure 5:**
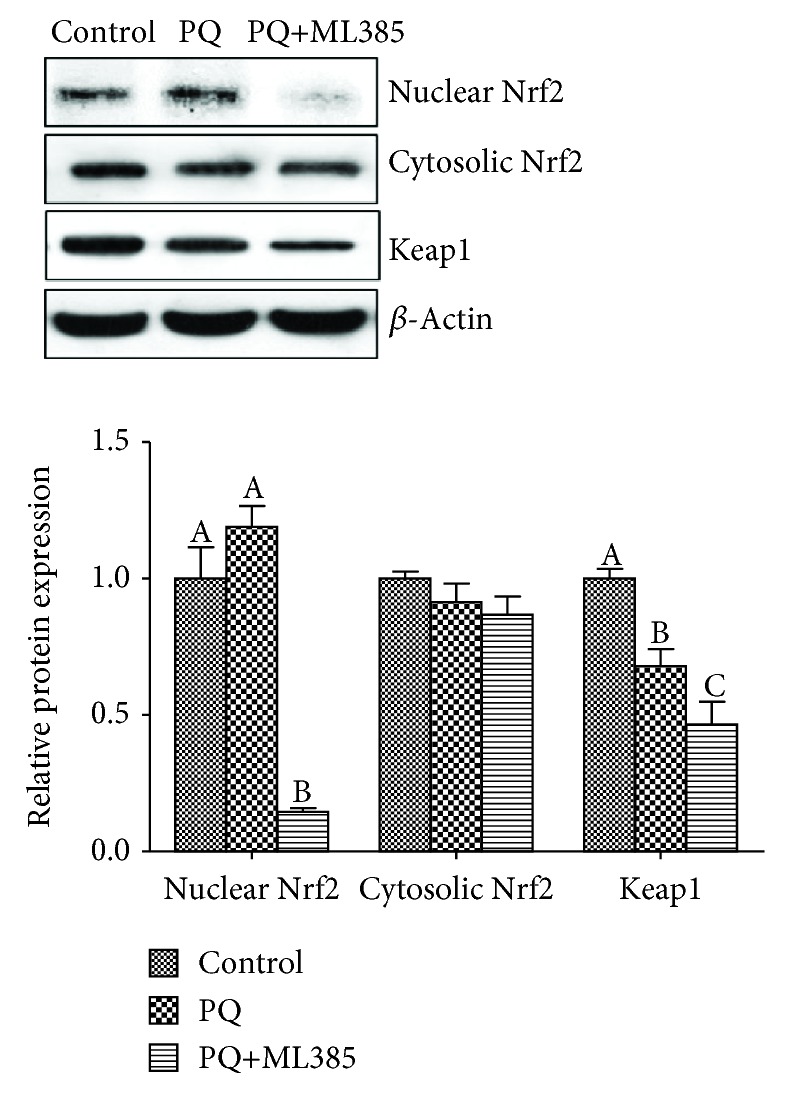
The representative western blot images and relative protein expressions of Nrf2 and Keap1 in IPEC-J2 cells. Cells were treated with DMEM-H medium (control) or with 70 *μ*M paraquat (PQ) or 1.9 *μ*M specific Nrf2 inhibitor (ML385) for 12 h. Data expressed as means ± SEM; *n* = 6 independent experiments. Different letters (A, B, and C) indicate significant differences (*p* < 0.05).

**Figure 6:**
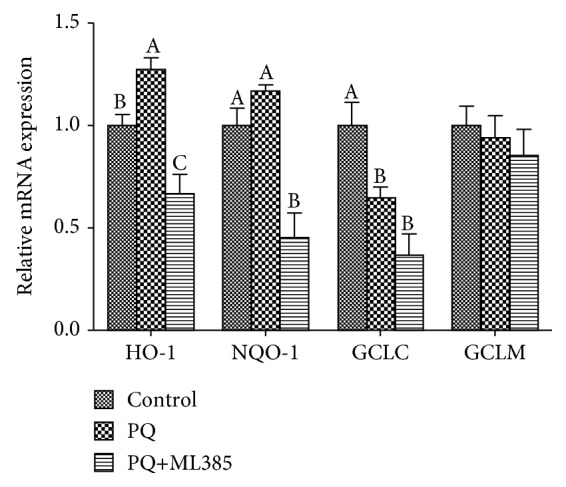
*HO-1*, *NQO-1*, *GCLC*, and *GCLM* mRNA expressions in IPEC-J2 cells. Cells were treated with DMEM-H medium (control) or with 70 *μ*M paraquat (PQ) or 1.9 *μ*M specific Nrf2 inhibitor (ML385) for 12 h. Data expressed as means ± SEM; *n* = 6 independent experiments. Different letters (A, B, and C) indicate significant differences (*p* < 0.05).

Compared with control cells, PQ treatments decreased Keap1 protein expression, and ML385 treatment decreased the protein levels of nuclear Nrf2 and Keap1 (*p* < 0.05). There was no difference in cytosolic Nrf2 protein expression among three treatments (*p* > 0.05) (Figure 5). For the downstream antioxidant enzyme, PQ induced an increasing of *HO-1* mRNA and a decreasing of *GCLC* mRNA expression. The mRNA expression of *HO-1*, *NQO-1*, and *GCLC* was decreased in response to the addition of both PQ and ML385 (*p* < 0.05) (Figure 6).

## 4. Discussion

The Nrf2 pathway is a hotspot in the field of antioxidant research in recent years. Targeting Nrf2-antioxidative stress signaling is an ideal strategy to prevent or treat oxidative stress-related diseases [12, 13, 19]. In addition, it has been proved by a large number of studies that some plant extracts could be potential inducers of the Keap1-Nrf2 pathway [20–25]. EUF has shown antioxidative activity and anti-inflammatory effects in piglets in the previous study; the present study is, therefore, focusing on its regulatory mechanisms involved in the Keap1-Nrf2 pathway.

Firstly, in the diquat-induced oxidative stress piglet model, dietary supplementation of EUF increased the protein expression of nuclear Nrf2 and Keap1. The Nrf2 pathway is critical for the regulation of intracellular reduction/oxidation (REDOX) status [26]. Under normal physiological conditions, Nrf2 is anchored in the cytoplasm by binding to Keap1, which is attached to the actin cytoskeleton, in order to maintain the cytoprotective enzyme and antioxidants at the basic expression levels and the cells in a stable state. When the production of free radicals is increased, Keap1 is converted to Keap1 thiol and/or Nrf2 is phosphorylated by protein kinase and released from Keap1 and translocates to the nucleus, thus activating the expression of antioxidant enzymes [27, 28]. It means that activated Nrf2 can protect cells against oxidants and electrophiles [29]. In the current experiment, however, diquat exposure decreased the protein expression of nuclear Nrf2 and Keap1 in the jejunum of piglets, which could be due to different doses of oxidants or the degree of oxidative stress in comparison to previously published research [26, 28, 30]. When piglets were challenged with 8 mg/kg body weight diquat for 1 week, the antioxidation system was completely destroyed [9], which might include disabled Nrf2. However, supplementation of EUF increased the nuclear Nrf2 protein expression, indicating the activation of the Nrf2 signaling pathway by EUF, which is consistent with previous research with other flavonoids [22, 24, 30–32].

Accordingly, Nrf2 has been demonstrated to regulate the expression of downstream antioxidant enzymes, including *NQO1*, *glutathione S-transferase*, *HO*, *gamma-glutamate synthetase*, *catalase*, *SOD*, *uridine diphosphate glucuronic acid base transferase*, *glutathione reductase*, and *GSH-Px*, in order to keep a normal REDOX state and reduce tissue damage by electrophilic reagent [33–35]. The present study observed that the mRNA expression of *HO-1* and *GCLC* was increased in the jejunum and ileum of diquat-challenged pigs supplemented with EUF. *HO-1* and *GCLC* were reported to be highly involved in antioxidant protection and suppression of inflammatory injuries in an *in vitro* human cell model and *in vivo* rat model [26, 36]. *GCL*, which consisted of a catalytic subunit (*GCLC*) and a modifier subunit, is the rate-limiting enzyme in GSH synthesis [37]. Therefore, the reduced mucosal GSSG concentration and GSSG/GSH ratio in EUF-supplemented pigs were likely due to the upregulated *HO-1* and *GCLC* expression.

The reduced gut morphology by diquat challenge due to the damage of oxidative balance has been demonstrated in the previous study [9, 38]. Diquat challenge could increase MDA concentration and inhibit the activities of antioxidant enzymes including SOD and GSH-Px in the blood [38, 39]. High intestinal oxidative stress in diquat-challenged piglets was indicated by significantly elevated levels of GSSG and GSSG/GSH ratio in the intestinal mucosa [40, 41]. Dietary supplementation of EUF improved the intestinal GSH REDOX state, as indicated by the reduced GSSG concentration and the ratio of GSSG to GSH in the jejunal and ileal mucosa. As discussed above, this was likely the result of the activation of genes involved in GSH biosynthesis and reduction [26]. Although no difference was observed in MDA concentrations in jejunal and ileal mucosa of pigs between basal diet and EUF diet, supplementation of EUF enhanced jejunal mucosa MDA concentration when compared with the diquat-challenged group. MDA represents one of end productions of lipid oxidation and is considered as an important marker of oxidative stress. Determination of MDA is the most frequently used method to evaluate lipid oxidation; however, it may not fully represent the complex oxidative status of animals, which includes more complicated products than lipid oxidation products [42]. More research is needed to fully evaluate the most representative markers of oxidative stress induced by diquat challenge.

To further demonstrate the role of the Nrf2 pathway in antioxidative stress in porcine enterocytes, a novel and specific Nrf2 inhibitor [43], ML385, was adopted in the current cell culture assay. Our original hypothesis was that ML385 could exacerbate oxidative stress of cells by interrupting the normal function of the Nrf2 pathway. In the present experiment, we observed that ML385 and PQ-treated cells had reduced protein expression of nuclear Nrf2 and Keap1 compared with only PQ-treated cells. ML385 could specifically interact with nuclear Nrf2 protein and then block Nrf2 transcriptional activity. In agreement with this statement, we also observed that cells treated with PQ and ML395 had downregulated downstream antioxidant enzyme (*HO-1*, *NQO-1*, and *GCLC*). Results of the current cell culture assays were in close agreement with previously published research that observed that ML385 reduced nuclear Nrf2 protein expression and downstream target gene expression [43, 44]. Overall, the important roles of the Nrf2 pathway in antioxidative function can be summarized: (1) cells treated with ML385 had decreased cell viability and increased cytotoxicity; (2) cells treated with ML385 had elevated AUC, intracellular GSSG concentration, and the ratio of GSSG to GSH; (3) ML385-treated cells had decreased *HO-1*, *NQO-1*, and *GCLC* mRNA expressions. Therefore, the Nrf2 signaling pathway played an important role in maintaining intracellular REDOX homeostasis [28] and functions as a ubiquitous, evolutionarily conserved intracellular defense mechanism to counteract oxidative stress [45].

In conclusion, the *in vivo* animal study observed that supplementation of EUF decreased GSSG concentration and the ratio of GSSG to GSH but increased the protein expression of nuclear Nrf2 and Keap1 as well as mRNA expression of *HO-1*, *NQO-1*, and *GCLC* in the small intestinal mucosa of diquat-challenged piglets. The *in vitro* cell culture assay confirmed that oxidative stress of paraquat-treated enterocytes was further enhanced when Nrf2 was inhibited by ML385. Results of the current research indicated that the Nrf2 signaling pathway played an important role in EUF-regulating oxidative stress in the intestine of piglets.

## Figures and Tables

**Table 1:** Small intestinal mucosal concentrations of SOD, MDA, GSH, and GSSG in piglets.

	Basal diet	Basal diet+diquat	EUF diet+diquat	*p* value
Jejunum				
SOD (U/mg prot)	15.671 ± 2.062^a^	7.678 ± 2.068^b^	13.263 ± 1.670^ab^	0.024
MDA (nmol/mg prot)	2.561 ± 0.256^ab^	1.698 ± 0.164^b^	3.356 ± 0.546^a^	0.014
GSH (mg/g prot)	0.315 ± 0.012	0.345 ± 0.014	0.354 ± 0.016	0.148
GSSG (mg/g prot)	0.029 ± 0.004^b^	0.076 ± 0.012^a^	0.032 ± 0.007^b^	0.001
GSSG : GSH	0.092 ± 0.005^b^	0.220 ± 0.020^a^	0.092 ± 0.003^b^	<0.001

Ileum				
SOD (U/mg prot)	13.623 ± 3.452	12.345 ± 2.087	14.568 ± 3.047	0.865
MDA (nmol/mg prot)	3.245 ± 0.541	3.546 ± 0.268	3.645 ± 0.317	0.759
GSH (mg/g prot)	0.298 ± 0.015	0.312 ± 0.042	0.309 ± 0.015	0.929
GSSG (mg/g prot)	0.018 ± 0.001^c^	0.084 ± 0.004^a^	0.037 ± 0.004^b^	<0.001
GSSG : GSH	0.062 ± 0.011^b^	0.269 ± 0.024^a^	0.119 ± 0.035^b^	<0.001

Values are the mean ± SEM; *n* = 8 per treatment group. ^a–c^Mean values sharing different superscripts within a row differ (*p* < 0.05).

**Table 2:** The mRNA levels of HO-1, NQO-1, GCLC, and GCLM in small intestinal mucosa of piglets.

	Basal diet	Basal diet+diquat	EUF diet+diquat	*p* value
Jejunum				
HO-1	1.000 ± 0.098^b^	1.121 ± 0.116^b^	3.717 ± 0.208^a^	<0.001
NQO-1	1.000 ± 0.087^a^	0.734 ± 0.066^b^	1.012 ± 0.038^a^	0.012
GCLC	1.000 ± 0.082^b^	1.133 ± 0.876^b^	1.674 ± 0.122^a^	<0.001
GCLM	1.000 ± 0.103	0.833 ± 0.116	1.167 ± 0.190	0.271

Ileum				
HO-1	1.000 ± 0.102^b^	0.553 ± 0.048^c^	1.897 ± 0.138^a^	<0.001
NQO-1	1.000 ± 0.043^a^	0.713 ± 0.067^b^	0.917 ± 0.087^ab^	0.021
GCLC	1.000 ± 0.059^a^	0.594 ± 0.098^b^	0.907 ± 0.082^a^	0.005
GCLM	1.000 ± 0.061^ab^	0.889 ± 0.055^b^	1.201 ± 0.070^a^	0.007

Values are the mean ± SEM; *n* = 8 per treatment group. ^a-b^Mean values sharing different superscripts within a row differ (*p* < 0.05).

## Data Availability

All original data used to support the findings of this study are available from the corresponding author upon request.

## Abbreviations

ARE: Antioxidant response element
AUC: Area under the curve
CAA: Cellular antioxidant activity
CCK-8: Cell counting kit-8
DCFH-DA: 2′,7′-Dichlorofluorescein diacetate
DMEM-H: High-glucose Dulbecco's modified Eagle's medium
EUF: *Eucommia ulmoides* flavones
GCLC: Glutamate cysteine ligase catalytic subunit
GCLM: Glutamate cysteine ligase modifier subunit
GSH: Glutathione
GSH-Px: Glutathione peroxidase
GSSG: Oxidized glutathione
HO-1: Heme oxygenase 1
LDH: Lactate dehydrogenase
MDA: Malondialdehyde
NQO-1: NAD(P)H:quinone oxidoreductase 1
Nrf2: NF-E2-related factor 2
PQ: Paraquat
REDOX: Reduction/oxidation
ROS: Reactive oxygen species
SOD: Superoxide dismutase.
